# Factorial structure of the Comprehensive Assessment of At-Risk Mental States in help-seeking individuals: mapping the structure and the prediction of subsequent transition to psychosis

**DOI:** 10.3389/fpsyt.2024.1381133

**Published:** 2024-05-24

**Authors:** Cristiana Montemagni, Anna Carluccio, Claudio Brasso, Flavio Vischia, Paola Rocca

**Affiliations:** Department of Neuroscience Rita Levi Montalcini, University of Turin, Turin, Italy

**Keywords:** help seeking, CAARMS, principal component analysis (PCA), disorganization, HoNOS, SOFAS

## Abstract

**Objectives:**

The aim of the current study was 3-fold: 1) to examine the factorial structure of the Comprehensive Assessment of At-Risk Mental States (CAARMS) in help-seeking individuals undergoing an assessment on suspicion of psychosis risk; 2) to investigate the association of CAARMS factors with functioning; 3) and to test the association of any derived factors with the longitudinal outcome of transition to psychosis.

**Methods:**

The study included 101 patients. First, a principal component analysis (PCA) was conducted using the Varimax rotation method. A minimum initial eigenvalues of greater than or equal to 1.0, analysis of Scree plots, percentage of variance explained by each component, reliability (Cronbach’s alpha) of factors above 0.7 and Parallel Analysis were the criteria used to determine the appropriate number of factors Second, Spearman correlations were run to analyze the relationship between CAARMS factors and sociodemographic and functional variables (i.e. age, schooling, Social and Occupational Functioning Assessment Scale-SOFAS- and Health of the Nation Outcome Scales-HoNOS- scores). Third, we performed a Logistic regression analysis to evaluate the association between baseline CAARMS factors and the risk of transition to psychosis at the 6-month follow-up.

**Results:**

A total of 101 consecutive patiens were recruited. We found that: 1) a 6 factor model solution as the most appropriate, jointly accounting for 65% of the variance; 2) factors 1 (“negative-interpersonal”), 2 (“cognitive-disorganization”), 3 (“positive”), and 4 (“motor-physical changes”) were negatively correlated with SOFAS total score; factors 1, 2, and 3 showed positive correlations with HoNOS total score; factors 2 and 3 present similar patterns of correlations, factor 3 manifesting the strongest association with HoNOS symptoms, HONOS and SOFAS total score. Both factors 5 and 6 show significant associations with HoNOS behavioral impairment; 3) after 6 months 28 participants (30.1%) converted to psychosis. Factors 2 and 3 were positively associated with the risk of transition to psychosis; whereas, the factor 5 (“affective factor”) was negatively associated with the outcome variable.

**Conclusions:**

It is thus crucial to recognize the type and severity of psychopathology in help-seeking individuals in order to intensive clinical monitoring of subclinical psychopathology risk profiles, and design specific care pathways.

## 1 Introduction

Primary psychosis usually arises from earlier stages that already indicate a requirement for care, and tends to coincide with other comorbid disorders and functional impairment. The concept of early intervention in psychosis has laid the groundwork for the development of clinical staging: this model aims to determine an individual’s placement on a continuum of illness, with the primary objective being to offer a more precise roadmap for treatment decisions, prognosis, and outcome prediction (1, 2). Interventions implemented during the subthreshold symptoms phase of disorder not only decrease the risk of transition for a minimum of 1–2 years, but also enhance functional outcomes (1, 2).

Over the last two decades, a critical clinical and research issue has been to define reliable and validated criteria to identify help-seeking subjects with subthreshold positive psychotic symptoms at high risk of developing a psychotic disorder such as the at-risk mental state (ARMS) (3) or clinical high-risk (UHR) (4).

These criteria comprise a combination of trait and state risk factors that allow the identification of individuals with an enhanced 36% risk of developing a psychotic illness within a year (5, 6), although declining transition rates to 15% have been found in specialized early intervention settings (7–9).

Even though the majority of individuals with ARMS will not actually transition to full-blown psychosis or even remit from an ARMS state, they manifest mental difficulties that are distressing and disabling *per se*, such as negative symptoms and persistent depressive and anxiety features that are associated with functional deficits at baseline and follow-up (9, 10). Indeed, psychiatric symptoms other than subthreshold psychotic ones represent the most common subjective distress eliciting the search for mental help in the general population (11–14).

In this scenario, a dimensional approach defining the extent to which, i.e., “how much”, a specific characteristic occurs could be a suitable method to interpret the proteiform phenomenology in ARMS help-seekers and to identify, describe, and map the emergent needs of this population. Indeed, it would serve as both an alternative and complement to the mainstream categorical approach based on all-or-nothing conditions determined by the “presence/absence” of specific symptoms, e.g., perceptual aberrations. This is already possible since the first and most widely used instrument, i.e., the Comprehensive Assessment of At-Risk Mental States [CAARMS] (15), allows one not only to rate positive symptoms (disorders of thought content, perceptual abnormalities, and disorganized speech) to classify the young person as non/UHR/affected by psychosis, but also to evaluate other valuable psychopathological domains such as cognitive-attentional changes, emotional disturbances, negative symptoms, impaired tolerance to normal stress, impulsive behaviors, behavioral changes, motor and physical changes, and general psychopathology, which appear to be associated with the emerging disability of young help-seekers (15). In detail, the CAARMS enables the assessment of subclinical symptoms that are below the sensitivity threshold of commonly used psychopathological scales. From this point of view, the exploration of the dimensional structure of the CAARMS might represent a useful way to describe the subclinical symptomatology in the UHR population (16–19).

To our knowledge, only three studies have explored the dimensional structure of CAARMS to better understand the UHR subjects’ psychopathology, suggesting the presence of three (17) or five (18, 20) factors.

In light of this, the aim of the current study was threefold (1): to examine the factorial structure of the CAARMS in help-seeking individuals undergoing an assessment on suspicion of psychosis risk, for an accurate representation of sub-threshold clinical manifestation in UHR (2); to investigate the association of CAARMS factors with other important variables such as global functioning (3); and to test the association of any derived factors with the longitudinal outcome of transition to psychosis, operatively defined as daily positive psychotic symptoms lasting longer than 1 week (6, 21).

## 2 Methods

### 2.1 Subjects and procedures

The present study includes all consecutive help-seekers on suspicion of psychosis risk referred to the Struttura Complessa Psichiatria Universitaria, Dipartimento di Neuroscienze e Salute Mentale, Azienda Ospedaliero-Universitaria “Città della Salute e della Scienza di Torino,” Turin, Italy, in the period January 2020–December 2023, for assessment and diagnosis, within the project named “Modello di riconoscimento e cura delle persone con esordio psicotico o ad alto rischio di psicosi nei giovani tra i 14 e i 30 anni, con un approccio fortemente orientato alla prevenzione” (“Model of recognition and care of people with onset psychotic or at high risk of psychosis in young people aged between 14 and 30 years, with a strongly oriented approach to prevention”).

Help-seekers were mainly sent by general practitioners, child neuropsychiatry centers, psychology/adolescent centers, other mental health services, hospital emergency rooms, school and college counselors, relatives, and self-referral. A very low threshold for the referral from any potential sender was chosen to favor the most inclusive accessibility.

Eligible patients fulfilled the following inclusion criteria (1): age between 14 and 30 years (2); seeking help for a mental distress with onset within the previous 12 months in association with substantial psychosocial functional decay [>25% decrease in the Global Assessment of Functioning (GAF) in the past 12 months] (3); fluent in Italian; and (4) being able to complete a full questionnaire. Exclusion criteria were the following (1): history of frank psychotic episodes (2); history of treatment with antipsychotics (3); substance abuse or dependence in the past 6 months (4); no consent from the adolescent or his/her parents (5); known intellectual disability (IQ < 70); and (6) anamnesis positive for a severe head injury (coma ≥ 48 h), neurological disorders, or any other medical condition associated with psychiatric symptoms. The presence of psychiatric comorbidity and substance use disorders (SUDs) was assessed using the SCID-5-TR.

All participants volunteered for the study and gave their written informed consent prior to participation. For individuals under 18, in addition to the informed consent, parents were informed and gave their written consent. The study complies with the Declaration of Helsinki and was conducted according to ethics committee approval (protocol number: 0057625).

Two experienced psychiatrists (AC and CM) conducted a semistructured interview to collect demographic and clinical data (age, gender, and years of education) and administered CAARMS, Social and Occupational Functioning Assessment Scale (SOFAS), and Health of the Nation Outcome Scales (HoNOS). The CAARMS rating was independently conducted by the two psychiatrists in the assessment of 25 individuals. The ICC for the CAARMS-K total score was 0.89, and those for the seven subscales (positive symptoms, cognitive changes, emotional disturbance, negative symptoms, behavioral change, motor/physical changes, and general symptoms) showed acceptable results (0.75, 0.74, 0.71, 0.68, 0.79, 0.61, and 0.85, respectively). To reduce inter-rater variability, all interviewers met for training workshops before the study began. The training procedure consisted of didactic sessions, observation, and supervised practice. In addition, procedure manuals and web-based instructional videos were always available for all interviewers.

### 2.2 Measures

The CAARMS is a semistructured interview developed at the Personal Assessment and Crisis Evaluation (PACE) Clinic in Melbourne and designed to assess prodromal symptoms (15). It consists of 27 items [each one calculated in terms of frequency/duration (0–6) and intensity (0–6)], which are grouped into seven subscales: (a) positive symptoms; (b) cognitive change, attention, and concentration; (c) emotional disturbances; (d) negative symptoms; (e) behavioral changes; (f) motor/physical changes; and (g) general psychopathology. In the present study, we used the approved Italian translation of the CAARMS (22), which showed good to excellent inter-rater reliability in Italian clinical samples (22–24).

The SOFAS is a 100-point single-item scale administered to assess the individual’s level of social and occupational functioning across a continuum ranging from optimum functioning to important functional impairment (25). The SOFAS is based on the GAF scale, but aims to separate functioning from symptoms.

The HoNOS is an observer-rated scale that covers psychological and behavioral symptoms and organic and social problems, and it has been developed for use with general psychiatric patients. It is composed of 12 items each scored 0 ± 4, yielding a total score in the range of 0 ± 48 (26). Higher scores indicate greater levels of impairment.

### 2.3 Procedure

Participants were re-interviewed 6 months after baseline assessment.

### 2.4 Data analysis

Statistical analysis was performed using SPSS Statistics (IBM) 28.0 with a critical *p*-value of 0.05. Mean ± standard deviation (SD) and percentages were calculated.

To examine the CAARMS underlying structure in the data from a help-seeking sample at first presentation, a principal component analysis (PCA) was conducted using the Varimax rotation method. PCA enabled researchers to reduce the number of dimensions in complex datasets. The principal components are ordered by their eigenvalues, which represent the variance in all the variables accounted for by a component. Only components with an eigenvalue > 1.0 were saved for subsequent analyses. After the first extraction, a varimax rotation of the components was performed.

There is no single optimal way of performing rotations. Our choice of using the varimax procedure was influenced by the purpose of this study—to identify distinct categories of symptoms that will elucidate the clinical picture of help-seeking individuals. Thus, a statistical technique that achieves the aim of making the pattern of loadings clearer and more definite (27–30) appeared more appropriate. In varimax rotation, the total amount of variation explained does not change and the components are adjusted in a way that makes the loadings either high positive (or negative) or zero, while keeping the components uncorrelated or orthogonal. Moreover, as varimax rotation is probably the most common approach to performing rotations, its use allows comparison with previous studies (18, 31, 32).

The validity of the factor analysis was analyzed using Bartlett’s sphericity test and the Kaiser–Meyer–Olkin (KMO) coefficient, with *p* < 0.05 and KMO > 0.6 as the cutoff levels, respectively.

The reliability of the questionnaire and the produced factors was evaluated using Cronbach’s alpha index, assuming 0.70 as the cutoff value (33).

Minimum initial eigenvalues of greater than or equal to 1.0 (KaiserGuttman rule) (34), analysis of Scree plots (the point of inflection on the scree plot), percentage of variance explained by each component (only factors that explained an additional 5% of the proportion of variance were retained), reliability (Cronbach’s alpha) of factors above 0.7, and parallel analysis (35) were the criteria used to determine the appropriate number of factors (36–38). The Horn’s parallel analysis is an inferential method that produces a huge number of random correlation matrices with the same number of variables and sample size as the actual matrix, and then makes comparisons between the mean eigenvalues from the random correlation matrices and the eigenvalues from the real data correlation matrix. Previous studies have shown that this analysis is the most accurate rule for identifying the correct number of factors (39, 40).

To be included in a given factor, an item had to possess a factor loading greater than 0.45 in its factor (41). If two items crossloaded to more than one factor with loadings >0.45, then all those were chosen and the item was considered to load on multiple factors. If only loadings below 0.45 exist, then all those >0.30 were chosen and the item was considered to load on multiple factors.

The normal distribution of the continuous variables was verified with the Kolmogorov–Smirnov test.

As the explored variables were non-normally distributed, we run Spearman correlations between CAARMS factors and baseline demographic, behavioral, and functioning variables.

Lastly, we performed a logistic regression analysis using a forward procedure to evaluate the association between baseline CAARMS factors and the risk of transition to psychosis at the 6-month follow-up.

Variance inflation factor (VIF) was used to detect the amount of multicollinearity.

## 3 Results

### 3.1 Sociodemographic sample characteristics

Among the 128 individuals who accessed the recruitment center during the investigation period, 101 completed the 6-month assessment. Among them, 51 were men and 50 were women; aged 20.6 ± 5.1 years; with 12.5 average years (± 2.3 years) of formal education; 17 not (engaged) in education, employment, or training (16.7%); 24 employed (23.5%); and 61 students (59.8%). Most of the patients were unmarried (69.6%).

Drop-outs showed higher scores in the fifth factor (“affective factor”) as compared with the completers.

### 3.2 Analyses on the whole sample

#### 3.2.1 Principal component analysis

The factor analysis’ validity has been proved by the Bartlett’s Test of Sphericity, with highly significant results (*χ* ^2^ = 1,815.60, df = 378, *p* < 0.001), which showed adequate sampling and nonlinearity of factors. Moreover, the KMO measure of sampling adequacy > 0.5 (0.638) highlighted both that there were relationships between the components and that they were selected appropriately. Both findings prove that the variables entered were adequate for factor analysis (29, 42).

The overall Cronbach’s alpha index of the CAARMS was 0.870, which confirms that the questionnaire is reliable (value > 0.70), while the item-total corrections varied from 0.125 to 0.602, except for item 7.6, which showed a correlation of −0.41 (**Table 1**). Median and mode values are also provided (see **Table 2**). Out of the 28 items, 3 items had 4 (moderately severe) as the most recurring score (mode), 5 items had 3 (moderate), 4 items had 2 (mild), and 16 items had 0 (absent).

**Table 1:** Corrected item-total correlations of the 28 CAARMS items and Cronbach’s alphas if the item is deleted.

	Corrected item-total correlation	Cronbach’s alpha if the item is deleted
1.1 Unusual thought content	0.571	0.861
1.2 Non-bizarre ideas	0.555	0.862
1.3 Perceptual abnormalities	0.314	0.870
1.4 Disorganized speech	0.580	0.861
2.1 Subjective cognitive change	0.508	0.865
2.2 Observed cognitive change	0.529	0.864
3.1 Subjective emotional experience	0.593	0.861
3.2 Observed blunter affect	0.454	0.865
3.3 Observed inappropriate affect	0.514	0.863
4.1 Alogia	0.589	0.863
4.2 Avolition/apathy	0.591	0.861
4.3 Anhedonia	0.559	0.863
5.1 Social isolation	0.576	0.862
5.2 Impaired role function	0.602	0.861
5.3 Disorganizing/odd/stigmatizing behavior	0.592	0.862
5.4 Aggression/dangerous behavior	0.198	0.872
6.1 Subjective complaints of impaired motor functioning	0.435	0.866
6.2 Informant reported or observed changes in motor functioning	0.497	0.866
6.3 Subjective complaints of impaired bodily sensation	0.411	0.866
6.4 Subjective complaints of impaired autonomic functioning	0.314	0.869
7.1 Mania	0.231	0.870
7.2 Depression	0.130	0.873
7.3 Suicidality and self-harm	0.125	0.877
7.4 Mood swings/lability	0.171	0.872
7.5 Anxiety	0.291	0.869
7.6 Obsessive–compulsive symptoms	−0.041	0.878
7.7 Dissociative symptoms	0.391	0.867
7.8 Impaired tolerance to normal stress	0.575	0.862

**Table 2:** CAARMS item scores at first assessment (*n* = 101): descriptive statistics.

	Minimum	Maximum	Mean	SD	Median	Mode
1.1 Unusual thought content	0	6	2.13	1.902	2	0
1.2 Non-bizarre ideas	0	6	2.14	1.802	2	0
1.3 Perceptual abnormalities	0	6	1.63	1.985	0	0
1.4 Disorganized speech	0	6	1.52	1.59	1	0
2.1 Subjective cognitive change	0	4	1.95	0.976	2	2
2.2 Observed cognitive change	0	5	0.62	1.137	0	0
3.1 Subjective emotional experience	0	5	2.07	1.381	2	2
3.2 Observed blunter affect	0	4	1.18	1.231	1	0
3.3 Observed inappropriate affect	0	5	1.33	1.446	1	0
4.1 Alogia	0	3	0.73	0.985	0	0
4.2 Avolition/apathy	0	5	2.57	1.507	2	4
4.3 Anhedonia	0	4	2.25	1.306	3	3
5.1 Social isolation	0	5	2.17	1.281	2	3
5.2 Impaired role function	0	5	2.51	1.6	3	4
5.3 Disorganizing/odd/stigmatizing behavior	0	4	1.32	1.292	2	0
5.4 Aggression/dangerous behavior	0	5	2.03	1.418	2	3
6.1 Subjective complaints of impaired motor functioning	0	4	0.62	1.088	0	0
6.2 Informant reported or observed changes in motor functioning	0	3	0.39	0.811	0	0
6.3 Subjective complaints of impaired bodily sensation	0	5	0.91	1.442	0	0
6.4 Subjective complaints of impaired autonomic functioning	0	5	1.41	1.446	2	0
7.1 Mania	0	3	0.20	0.65	0	0
7.2 Depression	0	6	2.63	1.324	3	2
7.3 Suicidality and self-harm	0	6	2.32	1.855	2	2
7.4 Mood swings/lability	0	5	2.70	1.264	3	3
7.5 Anxiety	0	5	3.02	1.398	3	3
7.6 Obsessive–compulsive symptoms	0	5	1.04	1.489	0	0
7.7 Dissociative symptoms	0	5	1.39	1.382	2	0
7.8 Impaired tolerance to normal stress	0	5	2.91	1.457	3	4

CAARMS, The Comprehensive Assessment of At-Risk Mental States; severity scores: 0 = Absent; 1 = Questionable; 2 = Mild; 3 = Moderate; 4 = Moderately severe; 5 = Severe; 6 = Extreme (i.e., psychotic intensity).

A PCA of the 28 items identified produced eight distinct and interpretable factors with eigenvalues greater than 1, overall explaining 75.9% of the variance. However, the scree plot representing the eigenvalues of the factors ordered by magnitude demonstrated a sharp point of inflection after the first seven factors. Of these, only the first six factors accounted for more than 5% of the variance. This finding was confirmed by the Horn’s parallel analysis (35), which showed only six components with eigenvalues exceeding the corresponding criterion values for a randomly generated data matrix of the same size. Taking these results into account, the findings of this study provide stronger support for a six-factor model solution as the most appropriate, jointly accounting for more than 65%, which is considered to be an acceptable result. The seventh factor (with modest loading on 5.4 and 7.4 items) explained 4.97% of the variance; the eighth one (with modest loadings positively on 7.8 and negatively on 3.2 items) explained only 4.75% of the variance. Both the seventh and eighth factors were excluded after examination of the parallel analysis.

**Table 3** presents the rotated component matrix of the CAARMS, percent variances explained, and items loading on each factor.

**Table 3:** Component loadings for the 28 CAARMS items.

	CAARMS factors					
	1	2	3	4	5	6
4.3 Anhedonia	0.834					
5.2 Impaired role function	0.772					
5.1 Social isolation	0.740					
4.2 Avolition/apathy	0.733					
7.8 Impaired tolerance to normal stress	0.649					
3.1 Subjective emotional experience	0.629					
5.3 Disorganizing/odd/stigmatizing behavior	0.599	0.451				
2.2 Observed cognitive change		0.866				
1.4 Disorganized speech		0.804				
7.1 Mania		0.735				
2.1 Subjective cognitive change		0.729				
4.1 Alogia		0.608				
1.1 Unusual thought content		0.530	0.518			
1.3 Perceptual abnormalities			0.800			
6.3 Subjective complaints of impaired bodily sensation			0.727			
1.2 Non-bizarre ideas	0.498		0.581			
6.1 Subjective complaints of impaired motor functioning				0.810		
6.2 Informant reported or observed changes in motor functioning				0.765		
3.3 Observed inappropriate affect				0.693		
7.3 Suicidality and self-harm					0.788	
7.7 Dissociative symptoms					0.725	
7.2 Depression					0.591	
6.4 Subjective complaints of impaired autonomic functioning						0.796
7.5 Anxiety						0.651
7.6 Obsessive–compulsive symptoms						0.613
5.4 Aggression/dangerous behavior						
7.4 Mood swings/lability						
3.2 Observed blunter affect	0.493					

Extraction method: principal component analysis. Rotation method: Varimax with Kaiser normalization. All loadings greater than 0.45 are reported Explained variance (extraction sums of squared loadings): Whole sample: Total = 65% (factor 1 = 16.9%; factor 2 = 14.17%; factor 3 = 9.77%; factor 4 = 8.55%; factor 5 = 8.26%; factor 6 = 7.4%).

In detail, the first factor explained 16.9% of the variance and included nine items (“anhedonia”, “impaired role function”, “social isolation”, “avolition”, “impaired tolerance to normal stress”, “subjective emotional experience”, “disorganized behavior”, “non-bizarre ideas”, and “observed blunted affect”), corresponding to a “negative-interpersonal factor” (**Table 3**). A second factor explained 14.17% of the total variance and was composed of seven items (“observed cognitive change”, “disorganized speech”, “mania”, “subjective cognitive change”, “alogia”, “unusual thought content”, and “disorganized behavior”), identifying a “cognitive-disorganization” factor. A third factor explaining 9.77% of the variance included four items (“perceptual abnormalities”, “subjective complaints of impaired bodily sensations”, “non-bizarre ideas”, and “unusual thought content”), representing a “positive” factor. A fourth factor explained 8.55% of the variance and included three items (“subjective complaints of impaired motor functioning”, “informant reported or observed changes in motor functioning”, and “observed inappropriate affect”), representing the “motor-physical changes” factor. Factor 5 explained 8.26% of the total variance and was defined by high loadings on three items (“suicidality and self-harm”, “depression”, and “dissociative symptoms”), reflecting a “mood-affective-emotional” domain. A sixth factor explained 7.4% of the variance and comprised three items (“subjective complaints of impaired autonomic functioning”, “anxiety”, and “obsessive–compulsive symptoms”). Factor complexity was observed; more than one item cross-loaded on more than one factor (item “disorganized behavior” on factors 1 and 2 and item “unusual thought content” on factors 2 and 3).

#### 3.2.2 Associations between CAARMS dimensions and demographic-functioning dimensions at baseline

**Table 4** presents the correlations between the CAARMS factors and baseline demographic, behavioral, and functioning variables. The level of statistical significance was set at *p* < 0.01 to compensate for multiple testing.

**Table 4:** Spearman correlations between CAARMS factors and baseline demographic, behavioral, and functioning variables.

	CAARMS factors					
	1	2	3	4	5	6
Age	−0.212 (0.033)				−0.382 (<0.001)	−0.416 (<0.001)
Education					−0.465 (<0.001)	−0.355 (<0.001)
HoNOS behavior					0.232 (0.019)	−0.225 (0.023)
HoNOS cognitive, physical problems		0.280 (0.004)	0.205 (0.039)			
HoNOS symptoms		0.296 (0.002)	0.577 (<0.001)	0.214 (0.031)		
HoNOS environment			0.270 (0.006)			
HoNOS total	0.228 (0.021)	0.369 (−0.001)	0.433 (−0.001)			
SOFAS	−0.256 (0.009)	−0.297 (0.002)	−0.442 (<0.001)	−0.316 (0.001)		

p-value < 0.01. HoNOS, Health of the Nation Outcome Scales; SOFAS, Social and Occupational Functioning Assessment Scale.

Correlations with age and schooling were all negative as regards factors 5 and 6, with CAARMS factor 5 having the strongest association with education and CAARMS factor 6 with age.

As regards functioning dimensions, factors 1, 2, 3, and 4 were negatively correlated with SOFAS total score; factors 1, 2, and 3 showed positive correlations with HoNOS total score. Factors 2 and 3 present similar patterns of correlations, with factor 3 manifesting the strongest association with HoNOS symptoms and HoNOS and SOFAS total score. Both factors 5 and 6 show significant associations with HoNOS behavioral impairment.

#### 3.2.3 Associations between CAARMS dimensions and transitions to psychosis

Participants were evaluated 6 months after baseline to assess whether psychosis had occurred.

During this time period, 28 out of 101 completers (30.1%) converted to psychosis. Only 1 out of 27 subjects who dropped out at follow-up transitioned to psychosis (clinical information obtained from referral providers, i.e., general practitioners, child neuropsychiatry, psychology/adolescent centers, other mental health services, and school and college counselors).

**Table 5** summarizes the results of the logistic regression analysis related to transition to psychosis as the outcome: Exp (B) is equivalent to the odds ratio (OR), a measure of a relationship’s strength between the predictor and the binary outcome. We found that the second factor (“cognitive-disorganization”) (hazard ratio = 1.282, 95% confidence interval = 1.022–1.607) and the third factor (“positive”) (hazard ratio = 1.393, 95% confidence interval = 1.065–1.823) were positively associated with the risk of transition to psychosis, whereas the fifth factor (“affective factor”) (hazard ratio = 0.661, 95% confidence interval = 0.476–0.918) was negatively associated with the outcome variable.

**Table 5:** Multivariable logistic regression analysis with multicollinearity measures for the three factors significantly associated (predictor variables) to transition to psychosis (dependent variable) over a 6-month period of observation.

	beta	SE	*p*	Exp (*B*)	95% CI per Exp (*B*)	VIF	Tolerance
Factor 2	1.59	0.082	0.051	1.172	0.999–1.375	1.193	0.838
Factor 3	0.258	0.101	0.011	1.294	1.062–1.576	1.013	0.987
Factor 5	−0.673	0.150	<0.001	0.510	0.380–0.685	1.208	0.828

Nagelkerke R^2^ = 0.754; VIF, variance inflation factor.

The association remained substantially unchanged (hazard ratio = 1.172, 95% confidence interval = 1.001–1.375 for factor 2; hazard ratio =1.294, 95% confidence interval =1.062–1.576 for factor 3; hazard ratio = 0.510, 95% confidence interval = 0.380–0.685 for factor 5) after controlling for age and gender.

The Nagelkerke *R* ^2^ value (i.e., *R* ^2^ = 0.754) demonstrates the model’s good prediction performance: these three predictors would explain more than 75% of the risk of transition to psychosis when controlling for age and gender.

Factors 1, 4, and 6 were not significantly associated with transition.

## 4 Discussion

In the present study, we aimed to explore the dimensional structure of the CAARMS, in order to verify whether types of UHR symptoms, as assessed by means of the CAARMS, co-occur in a sample of help-seeking individuals on suspicion of psychosis risk, and whether these “groupings” of symptoms at baseline were associated with subsequent transition to a full-blown psychosis.

Several key findings emerged from our research.

First, the internal consistency of the scale was good, as the Cronbach’s alpha coefficients for the CAARMS total score and the coefficients for single item were acceptable (above 0.5).

Second, item distributions within this sample reveal that most patients received low ratings on most symptoms. Individual item means typically fell below scale midpoints, with subjects exhibiting mild symptom presentations and most subjects rated as entirely normal on several symptoms. Moreover, we found that some symptoms are inherently multifactorial, i.e., disorganized behavior sometimes loads on both negative and disorganized factors, and unusual thought content loads on both cognitive/disorganized and positive factors.

Third, factor analysis of the CAARMS extracted six factors in our sample. Previously, factor analytic studies on the CAARMS revealed three underlying factors (negative-interpersonal, communicational-cognitive-behavioral disorganization, and perceptual-affective instability component) (17) and five factors (negative, anxiety, disorganization/cognitive, self-harm, and manic dimension) (18, 20). Other studies conducted on a conceptually similar instrument, the Scale of Prodromal Symptoms (SOPS), have found a three-model solution (31, 32).

Discrepancies in findings between studies may be due to several factors that may affect the homogeneity of the sample, its representativeness of the help-seekers population, and the components extracted by factor analyses. Some of these factors are referral pathways and settings [international research protocol on the first episode prodrome (31) vs. early intervention in psychosis program (32) vs. public mental health service for youth (17, 18)], recruitment [four sites located in USA and Canada (29) vs. community-based catchment area (17, 18, 32)], and sample size [94 (31) vs. 30 (32) vs. 122 (18) vs. 223 (17)].

Factor 1, labeled “negative-interpersonal” factor accounting for almost 17% of the variance, included items describing a general reduction in the ability to engage with the environment with vitality (anhedonia and avolition-apathy) and maintain good social and role performance (impaired role function and social isolation) and daily situational affective coping (impaired tolerance to normal stress). Symptoms loading on this factor, and not elsewhere, are clearly primarily negative in nature. This factor resembles the negative factor identified by Raballo et al. (17) and Demjaha et al. (18) (negative-interpersonal component and negative dimension, respectively). However, unlike Demjaha et al. (18), avolition, blunted affect, social isolation, and anhedonia did not load together with depression on the same dimension, suggesting that in this sample, negative symptoms should be considered an “independent” dimension, clearly distinct from depression (that loads prominently on a separate factor, the fifth), and probably “primary” (not a cooccurrence of depression). These discrepant findings from the literature may reflect some methodological differences between the studies such as the use of different rating scales, definitions for depression and negative symptoms, sampling strategies, and definitions of the populations to be sampled. Moreover, as the data from the CAARMS in the present study were collected cross-sectionally, it is early to draw firm conclusions that the negative symptoms in our sample are of primary nature until these findings are further explored and replicated in future studies. These results suggest that the negative dimension is the most prominent help-seekers’ characteristic, and is consistent with findings from a prodromal sample using another psychopathological instrument for prodromal symptoms, the SOPS (31, 32).

Factor 2, named the “cognitive-disorganized” component, representing the second contributor (14.17%) to the total variance, included items that comprised a broad range of subtle disturbances affecting subjective and objective cognitive changes and disorganized speech and behavior. In the CAARMS, Cognitive Change is a subscale comprising impaired attention, thought block, and racing thoughts, whereas Disorganized Speech includes two additional aspects of formal thought disorder: circumstantiality and tangentiality. Thus, our “cognitive-disorganized” factor manifests some resemblance to the “communication-attention and concentration problems” and “disorganization/cognitive” dimension found in one of the SOPS studies (32), in the other CAARMS studies (18, 20), and also the disorganization dimension identified in studies of patients with established psychosis, which typically comprises formal thought disorder and attentional impairment (43). Indeed, the two symptoms most consistently related to disorganization in established schizophrenia—”odd or bizarre behavior” and ‘‘conceptual disorganization’’ (thought disorder)—load with the cognitive symptoms (subjective and objective cognitive change) in this sample, supporting the notion of a unique factor, i.e., the cognitive/disorganized factor.

Factor 3, named “positive” component and explaining almost 10% of the total variance, comprised positive symptoms—reality distortion dimension (perceptual abnormalities, unusual thought content, and non-bizarre ideas), similar to psychotic positive factor in other studies, i.e., “unusual thought content-perceptual abnormalities” in Hawkins (31) and “unusual thought content-suspiciousness” in Lemos et al. (32). Consistent with the evidence of heterogeneity of the positive symptoms, a cardinal symptom of its domain, unusual thought content, also loads on the cognitive/disorganization factor, although our findings suggested that the psychotic and disorganization dimensions should be considered independent domains of psychopathology (44).

Interestingly, besides positive symptoms, one basic symptom (BS) (subjective complaints of impaired bodily sensations) was found to load on this factor. Unlike objectively observed and operationally defined positive and negative signs and symptoms, the BS items of the CAARMS (45, 46) assess subtle disturbances in drive, affect, thought, language, perception, motor and vegetative function, and stress tolerance from the subjective perspective reported by the help-seeker (47). Because BSs appear in the earliest prodromal stage, recognizing these symptoms promotes early detection and intervention (46). In this context, it is noteworthy that the factor consisting of BS was extracted, corroborating the recommendation that BS should be an important axis in evaluating prodromal individuals (46).

Factor 4, named the “motor-physical changes” component, explaining 8.55% of the total variance, included subjective (subjective complaints of impaired motor functioning) and objective impairment in motor functioning (observed changes in motor functioning) and one disorganized symptom (“observed inappropriate affect”).

It is apparently a quite heterogeneous factor; however, as both the items “subjective complaints of impaired motor functioning” and “observed changes in motor functioning” could be viewed as antecedents of “odd/stigmatizing behavior” (a disorganized symptom), we can hypothesize that these two symptoms loaded with another disorganized symptom (“observed inappropriate affect”) into a factor more homogeneous as appeared at first sight. Furthermore, the observed appropriateness of the affect is a direct manifestation of the subject’s vocal expressiveness, body language, and facial expressions in response to certain emotional stimuli. Therefore, impaired motor control might convey to the observer inappropriate affect that would be the result of a discrepancy between the affect experienced by the help-seeker and that which he or she is then actually able to express.

Factor 5, named the “mood-affective-emotional” factor, explaining 8.26% of the total variance, was exclusively made up of three symptoms, i.e., depression, self-harm/suicidality, and dissociative symptoms. Depressive symptoms are relatively common in the ARMS (48) and the nonspecificity of depression in our sample might reflect distress secondary to the recent onset of dissociative symptoms. Both depression and dissociative symptoms may trigger self-harm behavior. However, as our data were cross-sectional, we can only conclude that depression loaded together with self-harm and dissociative symptoms.

Factor 6, explaining 7.4% of the total variance, is more heterogeneous and includes one basic symptom (consisting of subjective complaints of impaired autonomic functioning) and two psychopathological symptoms (anxiety and obsessive–compulsive symptoms). Again, the cross-sectional design of the study does not allow us to ascertain if anxiety (free-floating and somatic and obsessive somatic ruminations) is a consequence of worry (and focalization) about impaired automatic functioning or if they simply occur together, loading on the same factor.

Fourth, cognitive-disorganization and positive factors presented similar patterns of correlations, with the latter manifesting the strongest association with impairment related to cognitive and physical problems (as indexed by HoNOS cognitive and physical problems) and problems related to symptoms (as described by HoNOS symptoms), besides HoNOS and SOFAS total score. It is not surprising, given that cognitive-disorganized and positive factors included items that can have an impact on the HoNOS and SOFAS total score. Cornblatt et al. (49) suggested that a sustained attentional impairment in CHR subjects might negatively affect social information processing, thus leading to an impairment in social interactions and the emergence of social difficulties and isolation. Poor global functioning has also previously been independently associated with the transition to psychosis in ARMS subjects (50). Therefore, the link between the severity of positive and disorganization/cognitive factors and poor global functioning and the evidence that all these may predict the subsequent onset of psychosis sustain both the clinical relevance of the factors extracted from this study and the hypothesis that they may each be a prodromal phenomenon of a common underlying process that increases the risk of developing a psychotic disorder (18).

Moreover, there was also a significant but less prominent correlation between the mood-affective-emotional dimension and HoNOS behavior. It is not surprising that subjects with higher levels of depression, dissociative symptoms, and self-harms are more likely, *ipso facto*, to exhibit higher impairment in behavior.

Fifth, the rate of psychosis among help-seeking subjects in our sample (i.e., approximately 30% within 6 months) is two to three (51) and two orders of magnitude higher than in the same at-risk age group in the general population or in help-seeking in specialized early intervention settings (10.1%–17.9% at 6 months) (15, 50), respectively. This suggests that in our early detection and intervention program, we evaluate subjects at risk in prodromic phases that are somewhat closer to frank psychosis than desired.

Sixth, in line with previous studies (17, 18, 21, 49, 52), we found that both the disorganization/cognitive and positive factors were positively associated with subsequent transition to psychosis. It is possible that these symptoms, generally considered core psychopathological features of schizophrenia, are the phenomenological expression of an underlying neurodevelopmental perturbation that confers a particularly high risk for the disorder (53). On the other hand, the mood-affective-emotional factor, characterized by mild to moderate levels of depression and mild level of self-harm, showed a negative association with the longitudinal outcome of transition to psychosis, probably identifying a different psychopathological trajectory.

There are several limitations in this study that researchers must consider in the interpretation of our findings: first, the relatively small sample size to assess the psychometric properties of the CAARMS, especially the factor structure; second, the examination of CAARMS scores only at baseline, and not longitudinally, with an experimental design that could allow mapping the fluctuations of both CAARMS components and clinical symptoms over time; third, the need for contextualization concerning the setting and referral pathway, which may be different from other UHR services; fourth, the recruitment of voluntary subjects who provided informed consent to participate in this research study, i.e., a selection bias in favor of participants with higher cooperativeness and/or more intense help-seeking.

## 5 Conclusions

The aim of this study was to depict the clinical manifestations of help-seeking individuals at the time of their referral to our service by adopting a dimensional approach (along continuous coordinates) instead of a categorical one (such as UHR criteria). We found the CAARMS to have an underlying six-factor structure. It can be observed that factors 1, 2, and 3 are relatively homogeneous.

Moreover, our observation that disorganized/cognitive dimension is associated with transition to psychosis suggests that, in addition to attenuated positive symptoms, more attention should be paid to these features, especially when pronounced at first contact/referral. The relevance of those subclinical psychopathological dimensions as targets for intervention is also highlighted by their strong association with global functioning. It is thus crucial to recognize the type and severity of psychopathology in help-seeking individuals in order to design specific care pathways, intensive clinical monitoring of subclinical psychopathology risk profiles, and interventions suitable for this group, as suggested by the ITAlian partnership for psychosis prevention (ITAPP) (54), which highlights the necessity to provide multidisciplinary interventions tailored to meet individual needs. These interventions should be standardized across centers and for follow-up periods to be extended, as a different longitudinal risk of psychosis onset has been observed across the CHR clinical academic centers.

Implementing early and personalized interventions presents a challenge due to clinicians’ limited understanding of mental risk states and the scarce resources. For the future, it would be desirable to promote training to equip teams to identify and assess individuals in prodromal phases; raise awareness among the population regarding mental health; increase the implementation of psychosocial interventions in services; and advocate for a multidisciplinary and personalized approach for the individual.

Indeed, beyond identifying at-risk individuals, the implementation of personalized early interventions tailored to subclinical psychopathological dimensions is crucial. This proactive approach fosters more effective clinical management strategies.

The Canadian Treatment Guidelines for Individuals at Clinical High Risk of Psychosis recommended psychosocial interventions as the first-line treatment for CHR individuals (55). Specifically, psychoeducation and cognitive behavioral therapy (CBT) for ultra-high-risk subjects not only help improve subclinical psychotic symptoms and psychosocial functioning but also reduce the risk of progression to psychosis (56). Psychoeducation has gained prominence as a preferred therapeutic approach, with individuals expressing greater readiness to engage in it compared to alternative interventions (57).

## Data availability statement

The datasets presented in this article are not readily available because the full datasets contain identifying information, and data sharing is subject to facility guidelines. Requests to access the datasets should be directed to cristiana.montemagni@unito.it. and paola.rocca@unito.it.

## Ethics statement

The studies involving humans were approved by Research Ethics Committee AOU Città della Salute e della Scienza di Torino. The studies were conducted in accordance with the local legislation and institutional requirements. Written informed consent for participation in this study was provided by the participants or participants’ legal guardians/next of kin. Written informed consent was obtained from the individual(s), and minor(s)’ legal guardian/next of kin, for the publication of any potentially identifiable images or data included in this article.

## Author contributions

CM: Conceptualization, Data curation, Formal analysis, Methodology, Writing – original draft, Writing – review & editing. AC: Data curation, Methodology, Writing – original draft, Writing – review & editing. CB: Supervision, Writing – original draft, Writing – review & editing. FV: Investigation, Resources, Writing – review & editing. PR: Supervision, Writing – original draft.
